# BCG Vaccine-Induced Innate and Adaptive Pulmonary Immunity Correlating with Protective Efficacy Against *Mycobacterium tuberculosis* in the Lungs

**DOI:** 10.3390/vaccines13080876

**Published:** 2025-08-19

**Authors:** Mayank Khanna, Alistair J. Ramsay

**Affiliations:** Department of Microbiology, Immunology and Parasitology, Louisiana State University Health Sciences Center, and the Louisiana Vaccine Center, New Orleans, LA 70112, USA

**Keywords:** BCG, *Mycobacterium tuberculosis*, vaccination, pulmonary immunity, innate immunity, adaptive immune responses, immune correlates, biomarkers

## Abstract

**Background/Objectives:** Effective prophylaxis for *Mycobacterium tuberculosis* (Mtb) requires greater understanding of immune correlates of protection. With renewed interest in BCG as an Mtb vaccine, particularly via the intravenous (IV) route, our objective was to characterize both innate and adaptive immune correlates of vaccine-induced pulmonary immunity as potential biomarkers for protective efficacy in a murine model of Mtb infection. **Methods:** Mice were given BCG via different routes and some boosted with recombinant virus constructs encoding Mtb Ag85B. Responding innate lymphoid cell (ILC) populations, T cells and B cells were analyzed by fluorescence activated cell sorting (FACS) for surface markers and by intracellular cytokine staining or antibody ELISPOT. Some immunized mice were challenged with aerosolized Mtb and monitored for bacterial growth in the lungs and spleen. **Results:** BCG given IV, but not intranasally or subcutaneously, resulted in marked increases in IFNγ expression at 72 h by pulmonary CD49^+^ NK cells, CD69^+^ ILC1, and two ILC3 populations, NCR-ILC3 and LTi cells, the latter also producing IL-22. Pulmonary ILC2 populations in these mice had significantly increased IL-13 expression at 24 h compared to the other routes. Interestingly, high levels of NK cells and ILC1 expressing IFNγ and/or TNFα were sustained at 8 wk, with sustained expression of IL-17A by pulmonary NCR-ILC3 and pronounced tissue-resident and effector memory CD4^+^ and CD8^+^ T cell responses. Intranasal boosting with Ad-Ag85B enhanced these T cell responses and generated Mtb-specific pulmonary IgA and IgG B cells, correlating with significantly reduced bacterial loads following Mtb challenge. **Conclusions:** BCG given IV primed for both early and persistent pulmonary ILC1/ILC3 responses of a predominantly Th1/Th17-type profile along with local Mtb-specific memory T cell and B cell populations, correlating with enhanced protective efficacy. These are worthy of further study as compartmentalized biomarkers for effective vaccine-induced local immunity against Mtb.

## 1. Introduction

*Mycobacterium tuberculosis* (Mtb) is the causative agent of tuberculosis (TB) with an estimated 1.25 million TB-related deaths in 2023 [1]. Globally, the TB incidence rate and estimated mortality increased between 2019 and 2021, likely due to disruptions and restricted access to TB services arising from the COVID-19 pandemic, reversing years of decline between 2005 and 2019, and it has now regained its spot as the leading global cause of death from a single infectious agent [1]. Bacille-Calmette-Guérin (BCG), the only currently licensed vaccine against TB, has been administered intradermally at birth as part of the Expanded Programme on Immunization since 1974 with a global coverage of 90% [2]. BCG is effective in reducing disseminated TB in infants; however, protection against pulmonary TB varies from 0 to 80% in adolescents and adults [3]. A subunit vaccine booster comprising a modified vaccinia Ankara strain (MVA) construct expressing Mtb antigen 85A (Ag85A) enhanced durable circulating Ag85A-specific T cell responses but failed to provide protection beyond the limited efficacy of BCG alone when tested in a phase 2b efficacy trial [4,5,6]. Potentially more promising results have been generated with another subunit vaccine, M72/AS01_E_, comprising a recombinant fusion protein derived from two Mtb antigens (Mtb32A and Mtb39A) combined with AS01_E_ adjuvant [7], while there are at least eleven candidates currently in phase II or III clinical trials [1]. Truly effective prophylaxis against Mtb remains elusive however, with greater understanding of immune correlates of protection against TB required to address this goal.

It is widely accepted that the route of vaccination can determine the nature and magnitude of the resultant immune response. Renewed interest in BCG as an effective vaccine against Mtb was stimulated by the demonstration that intravenous (IV) immunization, rather than subcutaneous (SC), intradermal or intranasal (IN) delivery, significantly improved protection against pulmonary Mtb challenge in macaques [8]. IV delivery of BCG also induced substantially more antigen-responsive CD4^+^ and CD8^+^ T cells in bronchoalveolar lavage fluids and in lung lymph nodes compared to other routes, correlating with its protective efficacy [8]. While it is unlikely that neonates or individuals in low resource settings would be given IV BCG, this work provided an important proof-of-concept for immune mechanistic studies. A significant challenge for TB vaccine development is that vaccine-induced biomarkers in the circulation may be poorly representative of potentially important local effects within the lungs [9]. Indeed, recent work with BCG vaccine in the macaque model points to the potential importance of local immune responses in the airways for protective efficacy against Mtb [10,11,12,13]. We attempted to further define both innate and adaptive immune correlates of vaccine-induced protective efficacy in the lungs following delivery of BCG IV, or via SC or IN routes, and in combination with viral delivery vectors, in a murine model of Mtb infection.

In the present study, we focused on resident memory T cells (T_RM_), antibody-secreting B cells, and innate lymphoid cell (ILC) populations. Three major groups of ILC, each comprising different cell subsets, have been identified on the basis of their production of signature cytokines, their receptor profiles, and their phenotypic markers. Group 1 (ILC1) may be defined by their capacity to produce IFNγ and comprise at least two cell types, conventional NK cells and tissue-resident ILC1 [14]. Group 2 ILC (ILC2) are also tissue-resident cells, particularly in the gut and lungs [15], secreting IL-5 and IL-13 in response to IL-25 and IL-33 [14]. Group 3 ILC (ILC3) encompass several cell types including natural cytotoxicity receptor positive (NCR)-ILC3 and lymphoid tissue inducer (LTi) ILC3 that secrete IL-22 and IL-17 and are thought to have similar functionality to Th17 cells [14,16,17]. Better understanding of these local innate and adaptive immune responses, and any correlation with protective efficacy, will help to delineate biomarkers that could be indicative of the potential efficacy of vaccination strategies against Mtb.

## 2. Materials and Methods

### 2.1. Mice

Six- to eight-week-old specific pathogen-free female BALB/c mice were purchased from Charles River (Wilmington, MA, USA) and were housed in the AAALAC-accredited LSUHSC Animal Care Facility for the duration of the studies. Mtb-challenged mice were housed in a Biosafety Level 3 (BSL3) Laboratory operated as recommended by the Centers for Disease Control and Prevention and monitored by the Louisiana State University Health Sciences Center (LSUHSC) Institutional Biosafety Committee. All procedures were conducted under the guidelines of the Institutional Animal Care and Use Committee of LSUHSC in accordance with National Institutes of Health (NIH) guidelines for the care and use of laboratory animals.

### 2.2. Immunization

All invasive procedures, except IV immunizations, were performed under anesthesia with a mixture of ketaThesia (ketamine HCl 100 mg/mL, Butler Animal Health Supply, Dublin, OH, USA) and xylazine (10 mg/mL, Henry Schein, Mandeville, LA, USA) diluted 8-fold in phosphate-buffered saline (PBS, Invitrogen, Waltham, MA, USA). BALB/c mice were immunized with either IV, IN, or SC with 1 × 10^6^ colony forming units (CFU) of *Mycobacterium bovis* BCG strain TMC 1011, obtained from ATCC (Rockville, MD, USA, catalog #35734). For IV and SC delivery, BCG was prepared in 100 μL PBS and in 20 μL PBS for IN delivery. Controls were sham immunized with PBS. To evaluate innate immune responses, mice were euthanized at 24 h, 72 h or 8 wk post-immunization and spleens and lungs were harvested by gross dissection. For prime-boost studies, mice were boosted with IN at 6 wk post-BCG prime, with 5 × 10^7^ PFU of recombinant adenovirus (Ad) or recombinant MVA virus vectors encoding Mtb antigen 85B (Ag85B), generated as described elsewhere [18,19], and delivered in 20 μL PBS. Controls were boosted with PBS only. At 8 wk post-boosting, mice were euthanized, and spleens and lungs were harvested by gross dissection. Harvested organs were processed into single-cell suspension in complete medium for use in immune assays [18,19,20,21].

### 2.3. Intracellular Cytokine Staining (ICS)

Cytokine expression by mononuclear cells was measured by ICS and data were analyzed using a hierarchical gating strategy, which included cell surface markers, as shown for ILC in Figures S1 and S3 [22] and for T cells in Figure S2.

For the evaluation of innate lymphoid cell populations, 2 × 10^6^ mononuclear cells were added to 96-well round bottom plates (Sigma-Aldrich, St. Louis, MO, USA) and incubated at 37 °C with 5% CO_2_ for 2 h. GolgiPlug protein transport inhibitor (BD Biosciences, Franklin Lakes, NJ, USA) was added at 0.1 μg/well and cells were incubated for a further 4 h, washed once with FACS buffer (PBS containing 1% fetal bovine serum), and stained with LIVE dead eFluor 780 (ThermoFisher, Waltham, MA, USA) for 30 min on ice and then washed once with FACS buffer. To exclude mature hematopoietic lineages during ILC identification, a FITC-conjugated antibody lineage cocktail (BioLegend, San Diego, CA, USA) was included. This cocktail contains antibodies targeting CD3e (T cells), CD11b (myeloid cells), CD45R/B220 (B cells), Ly-6G/Ly-6C (granulocytes), and TER-119 (erythroid cells). Live lineage-negative cells were then analyzed to identify ILC subsets. For group 1 ILC and group 3 ILC staining, fluorescent cell-surface antibodies for lineage cocktail-FITC, CD196 (CCR6)-BV605, CD45-BV570, CD335 (NKp46)-BV711, CD69-PerCP (all from BioLegend) and CD49b-BUV395 (BD Biosciences) were used. For group 2 ILC staining, antibodies for lineage cocktail-FITC, IL-33Rα (ST2)-PE-Cy7 (BioLegend) and IL-25R-PE (Invitrogen) were added to cells and incubated for 30 min on ice. Cells were washed once with FACS buffer and fixed with CytoFix/Cytoperm (BD Biosciences) on ice for 20 min and then permeabilized using a Fixation/Permeabilization kit (BD Biosciences) at room temperature for 20 min. For ILC1 and ILC3 staining, fluorescent-labeled antibodies against IL-17A-PE/Dazzle 594, IFNγ-PE, Granzyme B-PE-Cy7, TNFα-PB (BioLegend) and IL-22-PerCP/Cy5.5 (ThermoFisher) were added to cells and incubated for 30 min on ice. For ILC2 staining, IL-13-eFluor 450 (ThermoFisher) and IL-5-APC (BioLegend) were added to cells and incubated for 30 min on ice. Stained cells were washed once with PBS then fixed in 1% formaldehyde in PBS. A total of 500,000 events were acquired using an LSR II (BD Biosciences) flow cytometer.

For T cell analyses, 2 × 10^6^ mononuclear cells were stimulated with purified protein derivative (PPD) of *M. bovis* (Statens Serum Institut, Copenhagen, Denmark) at a final concentration of 5 μg/mL and incubated at 37 °C in 5% CO_2_ for 2 h. GolgiPlug protein transport inhibitor was added at 0.1 μg/well and cells were incubated for a further 4 h. For staining, cells were washed once with FACS buffer, stained with LIVE dead Aqua dye for 30 min on ice, washed again with FACS buffer and then stained with CD3-APC eFluor-780 (Invitrogen), CD4-APC, CD8-PE-Cy7, CD11b-PB, CD44-BV570, CD62L-BV711, CD103-BV605, F4/80-PerCP (BioLegend) and CD69-BUV-395 (BD Biosciences) on ice for 30 min. Stained cells were washed once and fixed with CytoFix/Cytoperm on ice for 20 min. Fixed cells were permeabilized using a Fixation/Permeabilization kit at room temperature for 20 min. Following permeabilization, cells were incubated IL-2-PE (BD Biosciences), IFNγ-PerCP/Cy5.5, IL-17A-PE/Dazzle 594 and Granzyme-FITC (BioLegend) on ice for 30 min, washed once with PBS and fixed in 1% formaldehyde in PBS. A total of 200,000 events were acquired using an LSR II flow cytometer.

### 2.4. Antibody-Secreting Cell (ASC) ELISpot Assay

Assays for Ag85B-specific IgA, IgG1 and IgG2a antibodies were performed as described previously [21]. Briefly, 96-well MultiScreen IP plates were coated with 2 μg/mL recombinant Ag85B protein (BEI Resources: NR-14870) in 100 μL PBS overnight at 4 °C. Plates were washed 5 times with PBS and blocked with 200 μL 5% nonfat dry milk (Bio-Rad, Hercules, CA, USA) for 1 h at room temperature. Single cell suspensions at 2 × 10^5^ cells were seeded per well and incubated in an incubator at 37 °C with 5% CO_2_ for 6 h. Cells were discarded and plates washed 5 times with PBS and incubated with biotinylated goat anti-mouse IgA, IgG1 or IgG2a (Southern Biotech, Birmingham, AL, USA) diluted in PBS containing 1% FBS and 0.05% Tween-20 (Sigma-Aldrich) overnight at 4 °C. Plates were subsequently washed 5 times with PBS, and 100 μL streptavidin–alkaline phosphatase (Promega, Madison, WI, USA) diluted in PBS was added to each well and the plates were incubated for 2 h in the dark at room temperature. To develop spots, plates were washed 5 times with PBS and 100 μL BCIP/NBT substrate (Moss Inc., Pasadena, MD, USA) was added to each well and incubated for 20 min at room temperature. Plates were washed with water and allowed to dry completely prior to counting spots using an AID-ELISpot counter (AID, Strasburg, Germany). Data are presented as spot-forming cells (SFCs) per million cells ± SEM.

### 2.5. Aerosolized M. Tuberculosis Challenge and Determination of Bacterial Load

Six weeks after vaccination, mice were challenged with a 50–100 CFU aerosolized *Mycobacterium tuberculosis* H3Rv strain (ATCC No. 27294. Rockville, MD) in a certified Biosafety Level 3 facility using a GlasCol aerosol infection system (Terre Haute, IN, USA), as described previously [19,20,21]. At 6 wk following the challenge, mice were euthanized to harvest the lungs and spleens to determine bacterial loads. Harvested organs were homogenized, serially diluted and plated on Middlebrook 7H10 agar plates containing 10% oleic-acid albumin-dextrose-catalase (OADC—BD Sparks, MD, USA) and 2 μg/mL of 2-thiophenecarboxylic acid hydrazide (Sigma-Aldrich) to selectively inhibit growth of residual BCG vaccine. Plates were incubated at 37 °C for 21 d before colonies were counted.

### 2.6. Statistical Analysis

One-way ANOVA with Tukey’s correction for multiple comparisons was used to analyze statistical differences using GraphPad Prism software 9.0. Data are presented as mean value ± SEM. *p* values ≤ 0.05 were considered statistically significant.

## 3. Results

### 3.1. Intravenous Delivery of BCG Leads to Persistent Th1/17-Type Cytokine Responses Among Group 1 and 3 ILC and Sustained Expression of IL-13 by Group 2 ILCs in the Lungs

Limited information is available concerning the nature of innate immune responses to BCG immunization, particularly in pulmonary tissues. We therefore evaluated the development of different ILC populations following BCG delivery IV or via other routes. Initially, we developed a strategy to identify distinct ILC populations at different timepoints post-immunization as described in Materials and Methods. Mice were immunized with BCG via IV, SC or IN routes and sacrificed 8 wk later for harvest and assay of mononuclear cells.

**Figure 1:**
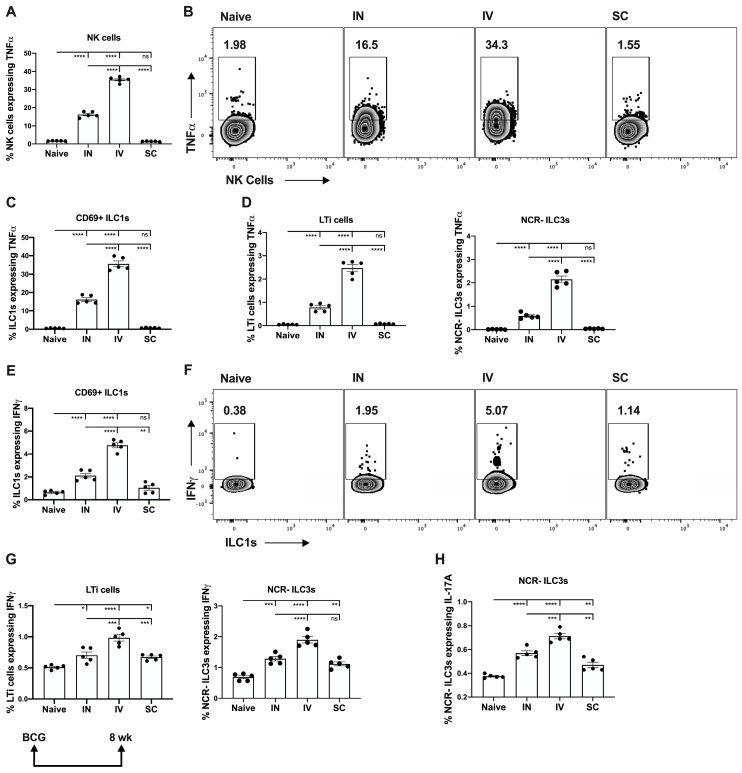
Mucosal ILC1/3 responses are sustained following BCG immunization via different routes. Mice were immunized with BCG via different routes and lung lymphoid cells were harvested 8 wk later and stained to characterize mucosal innate lymphoid cell (ILC) 1/3 cells as described in Materials and Methods. TNFα expression by (**A**,**B**) NK cells, (**C**) ILC1 or (**D**) Group 3 ILC, NCR-ILC3 and LTi cells, and IFNγ expression by (**E**,**F**) CD69^+^ ILC1 and (**G**) Group 3 ILC3, NCR-ILC3 and LTi cells, were determined by intracellular cytokine staining (ICS) and shown as a percentage of the parent cell populations. Representative flow cytometry plots are shown for (**B**) TNFα expression by NK cells and (**F**) IFNγ expression by ILC1 in lungs following immunization with BCG. Expression of Th17 cytokine IL-17A by NCR-ILC3 (**H**) is shown as a percentage of the parent cell population. Each solid circle on bar graphs is a data point representative of a single mouse within each group. Data are shown as mean values ± SEM. The statistical significance of differences in cytokine expression for different BCG immunization routes as marked were determined using one-way ANOVA with Tukey’s correction for multiple comparisons and are shown as * *p* < 0.05; ** *p* < 0.01; *** *p* < 0.001; **** *p* < 0.0001, ns, not significant.

Intravenous delivery of BCG resulted in a marked increase in TNFα expression by pulmonary natural killer (NK) cells (Figure 1A,B), CD69^+^ ILC1 (Figure 1C) and two group 3 ILC subpopulations, NCR-ILC3 and LTi cells (Figure 1D), compared to IN delivery, even at 8 wk post-immunization. There was no apparent TNFα expression by these ILC subsets after BCG was given SC. IFNγ expression by pulmonary CD69^+^ ILC1 (Figure 1E,F) and ILC3 (Figure 1G) was also significantly greater in the IV vaccine group, although there was no evidence for IFNγ expression in the CD49^+^ NK cell population at this timepoint). Intravenous BCG delivery also generated sustained expression of pulmonary NCR-ILC3 producing IL-17A, to a greater extent than IN or SC delivery (Figure 1H).

**Figure 2:**
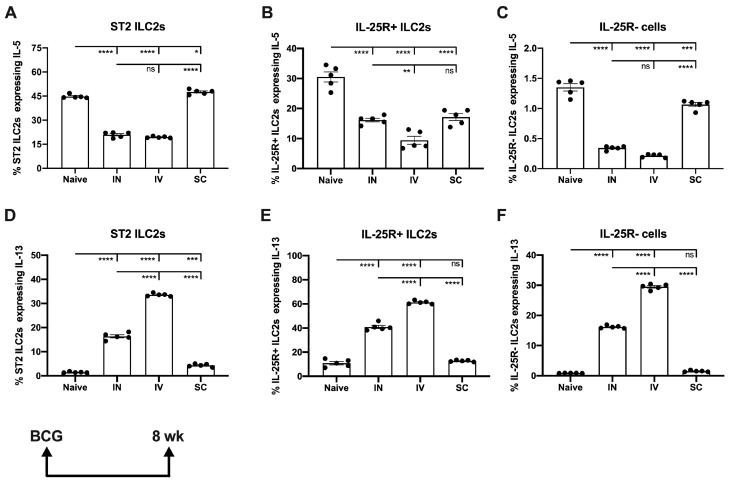
Mucosal ILC2 responses are sustained following BCG immunization via different routes. Mice were immunized and lung cells harvested as described in Figure 1 and were stained to identify ILC2 cells. Expression of IL-5 (**A**–**C**) and IL-13 (**D**–**F**) expression by tissue resident ST2+ ILC2 (**A**,**D**), migratory ST2-IL-25R+ ILC2 (**B**,**E**) and other innate lymphoid cells expressing Th2 cytokines ST2-IL-25R- cells (**C**,**F**), were determined by ICS and are shown as a percentage of parent cell populations. Each solid circle on bar graphs is a data point representative of a single mouse within each group. Data are shown as mean values ± SEM. The statistical significance of differences in cytokine expression for different BCG immunization routes were determined using one-way ANOVA with Tukey’s correction for multiple comparisons and are shown as * *p* < 0.05; ** *p* < 0.01; *** *p* < 0.001; **** *p* < 0.0001, ns, not significant.

Group 2 ILC may have similar functionality to Th2 cells. Using our gating strategy, both tissue resident ST2+ ILC2 (Figure 2A,D) and migratory ST2- IL-25R+ ILC2 (Figure 2B,E) were identified in the lungs, along with a further local subset of cells that were ST2-IL-25R- (Figure 2C,F) but expressed IL-5 and IL-13. Administration of BCG via the IV route led to significant decreases in IL-5 expression by group 2 ILC compared to SC delivery or in naïve animals (Figure 2A–C). Intranasal delivery of BCG also resulted in significantly decreased IL-5 expression by ILC2, with both ST2+ ILC2 and IL-25R- ILC2 expressing similar, reduced levels of IL-5 as seen following IV delivery (Figure 2A,C). However, sustained increases in IL-13 expression by ILC2 were seen in response to BCG given IV, with lesser increases seen following IN delivery (Figure 2D–F).

It is evident from these data that ILC may contribute to pulmonary immune responses for several weeks after immunization with BCG, particularly via the IV route.

### 3.2. BCG Generates Th1-Type ILC1 and ILC3 Responses in the Lungs Within 72 h Post-Immunization, Particularly via the IV Route

It was also important to evaluate ILC responses at early timepoints post-immunization, given that these cells are in a position to respond rapidly to tissue damage and may help to shape subsequent adaptive immunity. Mice were given BCG via different routes and sacrificed either 24 h or 72 h later for harvest of lung mononuclear cells for assay.

**Figure 3:**
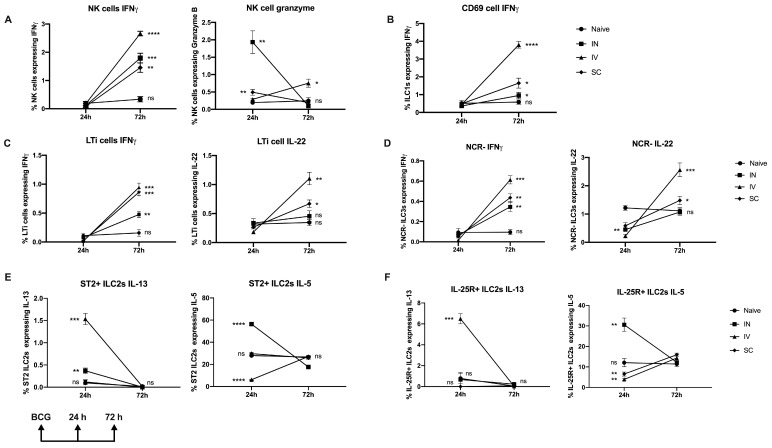
Mucosal ILC responses at early timepoints following BCG immunization via different routes. Mice were immunized and lung cells harvested, as described in Figure 1, at 24 h or 72 h later. Cells were stained to characterize cytokine expressing mucosal ILC, as described in Materials and Methods. Cytokine expression by (**A**) NK cells, (**B**) CD69+ ILC1 (**C**) LTi cells, (**D**) NCR-ILC3, (**E**) ST2+ ILC2, and (**F**) ST2-IL-25R+ ILC2, was evaluated by ICS at both timepoints. The statistical significance of differences between IV immunization and other routes at 72 h post immunization was determined using a paired Student’s *t*-test and are shown as * *p* < 0.05; ** *p* < 0.01; *** *p* < 0.001; **** *p* < 0.0001, ns, not significant.

Among group 1 ILC, IV BCG delivery resulted in increased IFNγ expression by pulmonary CD49b^+^ NK cells at 72 h at significantly greater levels than following IN or SC delivery (Figure 3A). Pulmonary NK cells harvested following IV immunization, but not via IN or SC routes, also showed significant increases in granzyme expression by 72 h (Figure 3A). IFNγ expression by pulmonary CD69^+^ ILC1 was also increased significantly by 72 h post-immunization in IV-immunized mice compared to IN or SC delivery (Figure 3B). Unlike at 8 wk (Figure 1C), there was no evidence for expression of TNFα by CD69^+^ ILC1 following IV delivery at this early timepoint.

Next, group 3 ILC were analyzed for IFNγ and IL-22 expression. Significant increases in IFNγ expression by pulmonary LTi-like cells were seen by 72 h after IV or SC immunization; however, IL-22 expression was much greater in the IV group (Figure 3C). Intranasal delivery of BCG led to less significant enhancement of IFNγ, but not IL-22, and expression by pulmonary LTi like cells by 72 h (Figure 3C). BCG given by any of these three routes resulted in increased expression of IFNγ by pulmonary NCR-ILC3 by 72 h, most significantly following IV delivery (Figure 3D), while all three routes also resulted in significantly reduced IL-22 expression at 24 h post-immunization compared to naïve controls (Figure 3D).

### 3.3. Differential Expression of IL-5 and IL-13 by Group 2 ILCs in Mice over 72 h Following IV Immunization with BCG

Group 2 ILC were also analyzed for Th2 cytokine expression in this system. Intravenous administration of BCG resulted in a significant increase in IL-13 expression by pulmonary ST2+ (Figure 3E) and IL-25R+ (Figure 3F) ILC2 by 24 h post-immunization, which had declined to levels observed in naïve mice by 72 h. Intranasal immunization resulted in lower levels of IL-13 expression by pulmonary ST2+, but not IL-25R+ ILC2 at 24 h post-immunization, which also declined to baseline levels by 72 h. In contrast, IV immunization resulted in reduced levels of IL-5 expression by both pulmonary ST2+ (Figure 3E) and IL-25R+ (Figure 3F) ILC2 by 24 h post-immunization compared to naïve mice, with expression increasing significantly by 72 h. Intranasal immunization, unlike IV delivery, resulted in significant increases in IL-5 levels in pulmonary ST2+ (Figure 3E) and IL-25R+ (Figure 3F) ILC2 at 24 h, which declined to baseline levels by 72 h.

### 3.4. Pulmonary Boosting with Ad-Vectored Vaccines Following IV BCG Prime Results in a Significantly Reduced Bacterial Load in the Lungs and Spleen Compared to BCG Prime Alone

**Figure 4:**
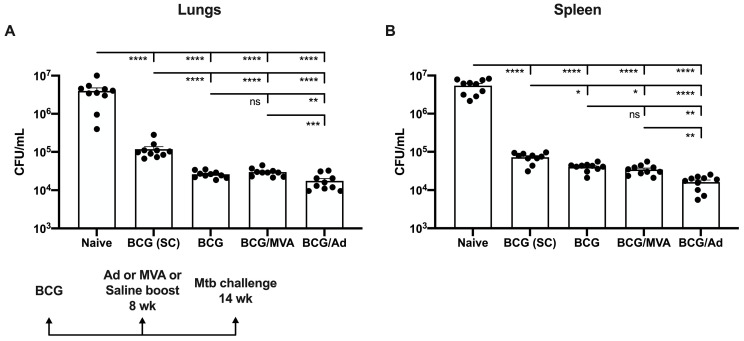
Bacillary loads in vaccinated mice following pulmonary Mtb challenge. Mice were immunized via IV or SC with BCG, some were boosted using IN with Ad or MVA constructs encoding Ag85B, and all were subsequently challenged with aerosolized Mtb H37Rv, as described in Materials and Methods. Lungs (**A**) and spleens (**B**) were examined for bacillary loads at 6 wk after challenge, as described in Materials and Methods. Each solid circle on bar graphs is a data point representative of a single mouse within each group. Data represent mean colony-forming units ± SEM for ten mice per group. Statistical analyses were performed using one-way ANOVA with Tukey’s correction for multiple comparisons. The significance of differences between vaccine groups are shown as * *p* < 0.05, ** *p* < 0.01, *** *p* < 0.001, or **** *p* < 0.0001, ns, not significant.

Having shown that IV BCG immunization results in a rapid onset of IFNγ expression by pulmonary group 1 and group 3 ILC that is apparently sustained for at least 8 weeks, along with high levels of TNFα and IL-17A expression, it was of interest to compare protection against pulmonary Mtb challenge following BCG administration. Mice were immunized via IV or SC with BCG, challenged with aerosolized pathogenic Mtb 6 wk later, and euthanized for enumeration of bacterial loads in lungs and spleens at 6 wk post-challenge. The data indicate that BCG-delivered IV (denoted as BCG in Figure 4) significantly reduces bacterial loads in lungs and spleens compared to BCG given SC (Figure 4A,B), with the observed four-fold reduction in lung titers being significantly greater than in spleens.

We also evaluated whether a booster vaccine could further enhance protection. Mice primed via IV with BCG were boosted with IN 8 wk later with either Ad-Ag85B or MVA-Ag85B, then challenged with Mtb and euthanized for enumeration of bacterial loads as above. Boosting with Ad-Ag85B, but not with MVA-Ag85B or PBS, resulted in a further two-fold reduction in bacterial loads in both lungs and spleens, indicating that an appropriate IN booster could enhance the protective efficacy of IV-delivered BCG (Figure 4A,B).

### 3.5. Intranasal Boosting with Ad-Vectored Vaccines Following IV BCG Prime Results in Pulmonary IgA and IgG Expression and Tissue-Resident and Effector Memory T Cell Activity

It was of interest to identify potential immune correlates of the protection observed following IV BCG delivery and also after subsequent IN boosting. Mice given BCG IV, and those primed with BCG IV and boosted via IN with Ad-Ag85B (BCG/Ad) or MVA-Ag85B (BCG/MVA) 8 wk later, were sacrificed at 6 wk after vaccination. Mononuclear cells from lungs and spleens were then isolated for use in assays for Mtb-specific pulmonary antibody responses and for tissue-resident CD44lo, CD103^+^, CD69^+^ T cell populations (T_RM_) and effector memory CD44hi T cell populations (T_EM_).

**Figure 5:**
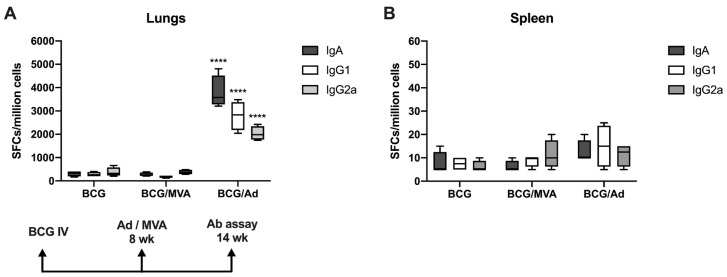
Mtb-specific mucosal antibody responses following BCG prime and viral vector boost. Mice were IV primed with BCG and IN boosted 8 wk later with MVA or Ad constructs encoding Ag85B, as described in Materials and Methods. Ag85B-specific antibody responses were measured in lungs (**A**) and spleens (**B**) by ELISPOT assays at 6 wk after boost. Data are shown as mean numbers of spot-forming cells (SFCs) per million cells ± SEM. The significance of differences in IgA, IgG1 and IgG2a responses between BCG and BCG/Ad vaccine groups were determined by one-way ANOVA with Tukey’s correction for multiple comparisons and are shown as **** *p* < 0.0001.

Strikingly, IN boosting with Ad-Ag85B, but not with MVA-Ag85B, resulted in significantly increased numbers of pulmonary B cells secreting Ag85B-specific IgA, IgG1 or IgG2a compared to IV BCG priming only, as measured in antibody-secreting cell (ASC) ELISPOT assays (Figure 5A). Ag85B-specific antibody-secreting B cells were not detected in the spleens of immunized mice, regardless of the vaccination route (Figure 5B), nor were they found in significant numbers in mice given Ad-Ag85B without BCG priming.

**Figure 6:**
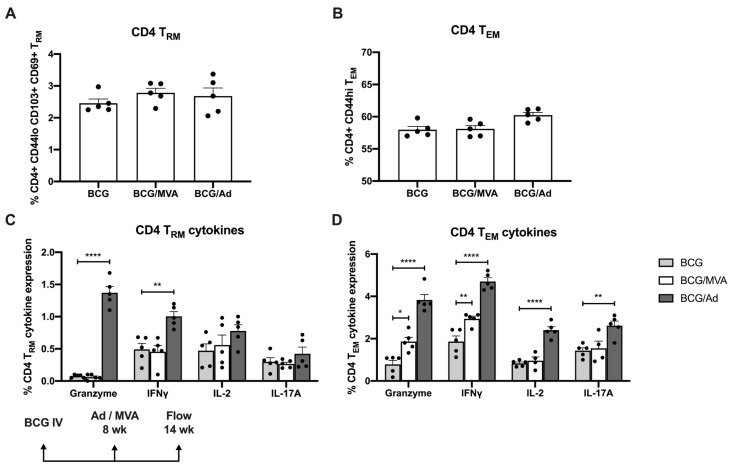
Expression of antimicrobial cytokines by Mtb-specific pulmonary CD4^+^ memory T cells following BCG/viral vector prime-boost vaccination. CD4^+^ T cells isolated from the lungs of mice immunized as described in Figure 4 were analyzed by flow cytometry at 6 wk post-boosting for surface expression of (**A**) resident memory (T_RM_) and (**B**) effector memory (T_EM_) cell markers. ICS was used to evaluate Mtb-specific cytokine expression by (**C**) CD4 T_RM_ and (**D**) CD4 T_EM_ cells. Each solid symbol on bar graphs is a data point representative of a single mouse within each group. Data are shown as mean values ± SEM. The significance of differences in cytokine expression between vaccine groups was determined using an unpaired Student’s *t*-test and are shown as * *p* < 0.05; ** *p* < 0.01; **** *p* < 0.0001.

**Figure 7:**
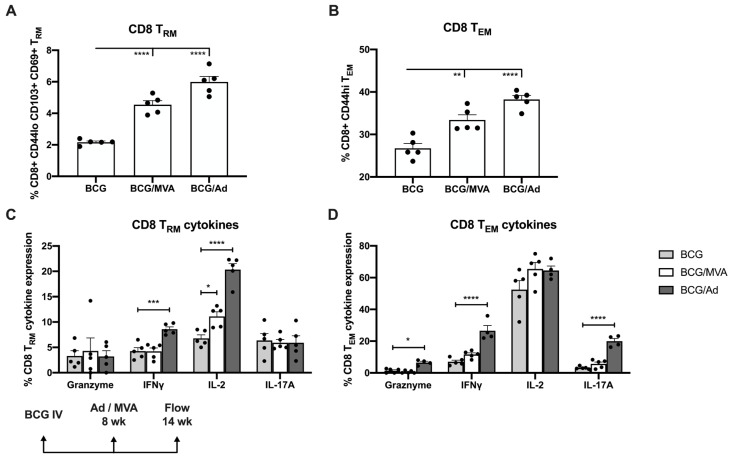
Expression of antimicrobial cytokines by Mtb-specific pulmonary CD8^+^ memory T cells following BCG/viral vector prime-boost vaccination. CD8^+^ T cells isolated from the lungs of mice immunized as described in Figure 4, were analyzed by flow cytometry at 6 wk post-boosting for surface expression of (**A**) resident memory (T_RM_) and (**B**) effector memory (T_EM_) cell markers. ICS was used to evaluate Mtb-specific cytokine expression by (**C**) CD8 T_RM_ and (**D**) CD8 T_EM_ cells. Each solid symbol on bar graphs is a data point representative of a single mouse within each group. Data are shown as mean values ± SEM. The significance of differences in cytokine expression between vaccine groups was determined using an unpaired Student’s *t*-test and are shown as * *p* < 0.05; ** *p* < 0.01; *** *p* < 0.001; **** *p* < 0.0001.

Each of these immunization strategies (ie. BCG, BCG/Ad-Ag85B, or BCG/MVA-Ag85B) generated strong pulmonary CD4 T_RM_ and CD4 T_EM_ responses (Figure 6A,B). However, priming with BCG IV followed by IN boosting with Ad-Ag85B generated significantly higher numbers of PPD-specific granzyme-positive and IFNγ-expressing CD4 T_RM_, and granzyme-positive and IFNγ, IL-2 or IL-17 expressing CD4 T_EM_, than either IV BCG alone or following boosting with MVA-Ag85B (Figure 6C,D). In terms of CD8^+^ T cell responses, IN boosting with either Ad-Ag85B or MVA-Ag-85B significantly elevated numbers of both CD8 T_RM_ and CD8 T_EM_ in the lungs, although this was more pronounced in those given Ad-Ag85B (Figure 7A,B), as were increases in PPD-specific pulmonary IFNγ and IL-2-expressing CD8 T_RM_, and granzyme-, IFNγ- and IL-17A-expressing T_EM_ (Figure 7C,D). Interestingly, CD4 and CD8 T_RM_ or T_EM_ cells were found only in very low numbers in the spleen following IV BCG.

Together, these findings show that Ad-vectored vaccines, when delivered as IN boosters following IV priming with BCG, enhance Mtb-specific pulmonary T and B cell responses that correlate with reduced bacterial loads in lungs and spleens following Mtb challenge. They also suggest that vaccine-induced pulmonary T_EM_ and T_RM,_ along with local IgA and IgG antibody responses, should be further studied as potential correlates of protection for targeting by vaccination strategies against Mtb.

## 4. Discussion

*Mycobacterium tuberculosis*, the causative agent of pulmonary TB, enters the body predominantly via aerosol droplets and deposits onto the alveolar surfaces of the lungs [23]. Improved understanding of pulmonary immune responses following infection or vaccination may therefore help to delineate biomarkers that could be used to monitor the potential efficacy of effective immunization strategies against Mtb [9,10,11,12,13,19,20,24]. We therefore attempted to characterize both innate and adaptive immune responses following BCG delivery by different routes, and in prime-boost combination with viral delivery vectors in a murine model of Mtb infection. Our findings show that IV delivery of BCG resulted in the development of pulmonary ILC1/ILC3 populations displaying predominantly Th1/Th17 cytokine profiles that apparently persisted for at least 8 wk after immunization. Mycobacterium-specific pulmonary T_RM_ and T_EM_ responses were also prominent in these animals, with both the local ILC and T cell populations correlating with significantly decreased bacterial loads in the lungs of Mtb-challenged mice. Local vaccine boosting with Ad-Ag85B further enhanced numbers of pulmonary CD4^+^ and CD8^+^ T_RM_ and T_EM_ and led to the development of Ag85B-specific IgA- and IgG-secreting B cells in the lungs, correlating with enhanced protection.

Recent evidence that IV delivery of BCG limits pulmonary Mtb infection in susceptible rhesus macaques has significant implications for vaccine design [8]. Using the Mtb challenge model, and in agreement with these findings in macaques, we observed significantly reduced bacterial loads in the lungs and spleens of mice that had been IV immunized with BCG compared to SC delivery, the gold standard route for immunization studies in the murine Mtb model. Adding an IN-delivered Ad-Ag85B booster at 8 wk after IV BCG priming further reduced bacterial loads in the lungs and spleen, unlike boosting with MVA-Ag85B. Intranasal but not IM boosting with an Ad-vectored vaccine encoding Ag85A following SC BCG priming was previously shown to enhance protection over that conferred by SC BCG alone [25]. Since Ad are primarily respiratory viruses, Ad-vaccine vectors would be expected to target the lungs, where Mtb primarily resides [26]. It should be noted that Ag85B, as expressed in our vaccine constructs, is just one of multiple immunogenic antigens expressed by Mtb. The inclusion of different or multiple such antigens in our constructs may generate broader protective immunity, at least in the case of adaptive responses. In any case, an important conclusion from our findings is the potential to enhance the level of protection observed following IV delivery of BCG.

Mice boosted with the Ad-Ag85B vaccine following IV BCG priming also developed Ag85B-specific IgA- and IgG-secreting B cells in the lungs, which appear to correlate with the decreased bacterial loads that we observed in the lungs of Mtb-challenged mice. The role of humoral immunity in TB control remains uncertain; however, B cell-deficient mice have increased susceptibility to infection with clinical Mtb strains [27], while Mtb-specific B cells appear to drive differentiation and accumulation of T follicular helper cells in granuloma-associated lymphoid tissues and influence their organization [28]. In a BCG challenge model, IgA deficient mice were more susceptible to infection than wild-type mice [29]. Furthermore, antibody responses to the Ag85 complex were associated with superior outcomes in an Mtb-infected patient cohort [30]. Specific antibody levels were also elevated in bronchoalveolar lavage fluids and plasma in macaques primed with BCG intravenously [8]. Antibody-mediated immunity against Mtb could include bacterial neutralization, enhanced inflammasome activation, complement activation, and enhanced NK cell activity [12,31,32], while local IgA antibodies generated following BCG immunization, along with polyfunctional Th17 cells, have recently been shown to correlate with protection against pulmonary Mtb challenge [12], as in the present study. While confirmatory studies will be needed to clarify a mechanistic role for specific antibodies in local protection, including B cell depletion and passive transfer experiments, there is increasing evidence for pulmonary antibody secreting B cells as biomarkers for protection against Mtb.

Intravenous BCG immunization also generated strong, mycobacterium-specific pulmonary T_RM_ and T_EM_ responses, correlating with protection in our model. Local boosting, particularly with Ad-Ag85B, significantly enhanced numbers of CD8^+^ T_RM_ and T_EM_, with pronounced enhancement of CD4^+^ T cell populations producing granzyme and IFNγ, and T_EM_ cells producing IL-17A. Lung-localized T_RM_ cells have been reported as strong correlates of protection against Mtb challenge in the abovementioned report of IV BCG immunization in rhesus macaques [8], and also following local IN or intra-tracheal delivery [33,34]. More recent studies have described an influx of polyfunctional T cells in the airways following IV BCG immunization, including CD4^+^ T cells producing TNF along with IFNγ or IL-17 [11] or Th1/Th17 cells [13]. In contrast, levels of circulating mycobacterium-specific T cells do not appear to correlate well with protection [9,13]. Thus, increasing evidence suggests that local polyfunctional memory T cells, including tissue-resident populations, may represent important correlates of protective immunity against pulmonary TB infection.

While many studies have focused on adaptive immune responses as potential biomarkers of protection against Mtb infection, relatively little is understood of the nature and involvement of innate immune responses, particularly in local pulmonary tissues. Here, we focused on ILC populations in pulmonary tissues at both early and later timepoints after BCG delivery by different routes. Interestingly, our findings suggest that pulmonary ILC may contribute to local immune responses in the lungs for at least several weeks after immunization. ILC represent a heterogeneous cell population having diverse roles in immunity and may represent innate counterparts of T cell sub-populations that respond swiftly to pathogenic tissue damage by secreting cytokines, responses that may also help to shape subsequent adaptive immunity [35]. Our data indicate that a variety of pulmonary ILC phenotypes are expressed at both early (24–72 h) and later (8 wk) timepoints after BCG immunization, particularly via the IV route.

Among group 1 ILC, early expression of IFNγ was markedly increased in both CD49b^+^ NK cells and CD69^+^ ILC1 when BCG was given IV compared to SC or IN, while NK cells also showed pronounced expression of TNFα and granzyme in the IV group. Interestingly, the SC route of BCG delivery that has served as a comparative standard for protection against Mtb in evaluations of a variety of other vaccination strategies failed to induce pulmonary NK cells expressing TNFα at the 8 wk timepoint. An extensive body of work has demonstrated a role for NK cells in controlling Mtb growth, including in human studies, attributed to their production of antimicrobial cytokines [36,37,38]. Significant increases in IFNγ and IL-22 expression were seen in group 3 ILC populations (i.e. NCR-ILC3s and LTi-like cells) at 72 h, most prominently following IV BCG. A Th1/Th17-type cytokine profile predominated among group 1 and group 3 ILC at 8 weeks after BCG immunization, and this was clearly most pronounced following IV delivery.

Group 2 ILC are thought to have similar functionality to Th2-type cells [17]. In our model, group 2 pulmonary ILC populations produced significantly less IL-5 at both 24–72 h and 8 wk after IV BCG immunization compared to the IN and SC routes. Sustained increases in IL-13 expression by these cells were also observed, with lesser increases seen following IN immunization. While the significance of this finding requires further investigation, IL-13 is known to modulate macrophage function associated with tissue repair and immune defense in addition to its role in B cell differentiation [39,40].

Since ILC are positioned to respond rapidly to tissue damage and may also help to shape subsequent adaptive immune responses, it is interesting that IV delivery of BCG resulted in a largely Th1/Th17 profile, particularly given the demonstrated protective efficacy of BCG when given by this route [8]. IL-17 expression has been shown to play a key role in regulating cell-mediated adaptive immunity in Mtb infection, promoting CD4^+^ T cell recruitment to pulmonary sites and accelerating pathogen clearance in BCG-immunized mice challenged with Mtb [41,42]. The spontaneous ex vivo production of IL-17A by pulmonary NCR-ILC3s in our study following IV BCG immunization illustrates a potential early source of this factor that could enhance local CD4^+^ T cell recruitment. In other recent studies using the mouse model, group 3 ILC accumulated rapidly in Mtb-infected lungs, coinciding with local accumulation of macrophages, while expansion of this population, along with local IL-17/IL-22 production, was important for the formation of protective lymphoid follicles within granulomas [43]. While it has also been reported that BCG can activate ILC1 and NK cells in lungs and lymph nodes when given IN [44], our finding that group 1 and group 3 ILC displaying predominantly Th1/Th17 cytokine profiles are present in pulmonary tissues, but not in the spleen, for at least 8 wk after IV BCG immunization is noteworthy, suggesting that these cells could also contribute to ongoing local immunity against mycobacterial infection. Studies are planned to determine if ILC populations, particularly ILC1/3 responses of this nature, persist beyond 8 wk and even post-challenge with Mtb.

## 5. Conclusions

Intravenous delivery of BCG resulted in both early and persistent ILC1/ILC3 responses in pulmonary tissues, along with local Mtb-specific CD4^+^ and CD8^+^ T_RM_ and T_EM_ responses, which correlated with enhanced protective efficacy in a mouse model of Mtb infection. Adaptive immune responses, including the development of pulmonary IgA and IgG B cell populations, followed local Mtb-specific vaccine boosting. These innate and adaptive responses are worthy of further study as compartmentalized biomarkers for effective vaccine-induced local immunity against Mtb.

## Data Availability

Raw data will be available upon suitable request to the corresponding author.

## Supplementary Materials

The following supporting information can be downloaded at: https://www.mdpi.com/article/10.3390/vaccines13080876/s1, Figure S1: Gating strategy for analysis of innate lymphoid cells; Figure S2: Gating strategy for analysis of memory T cells; Figure S3: Gating strategy for cytokine expression by ILC populations.

## Abbreviations

The following abbreviations are used in this manuscript:

Ad	adenovirus
Ag85B	antigen 85B of Mycobacterium tuberculosis
BCG	Bacille–Calmette–Guerin
ICS	intracellular cytokine staining
ILC	innate lymphoid cells
IN	intranasal
IV	intravenous
Mtb	mycobacterium tuberculosis
PPD	purified protein derivative
SC	subcutaneous
SFC	spot-forming cells
T_EM_	effector memory T cells
T_RM_	tissue resident memory T cells
