# Supplementary material for: BCG Vaccine-Induced Innate and Adaptive Pulmonary Immunity Correlating with Protective Efficacy Against Mycobacterium tuberculosis in the Lungs
Source: Vaccines (Basel). 2025 Aug 19;13(8):876. doi: 10.3390/vaccines13080876 (PMC12389847; doi:10.3390/vaccines13080876)
Supplement: Supplementary file 1 [file vaccines-13-00876-s001.zip › Figure S1.pdf]

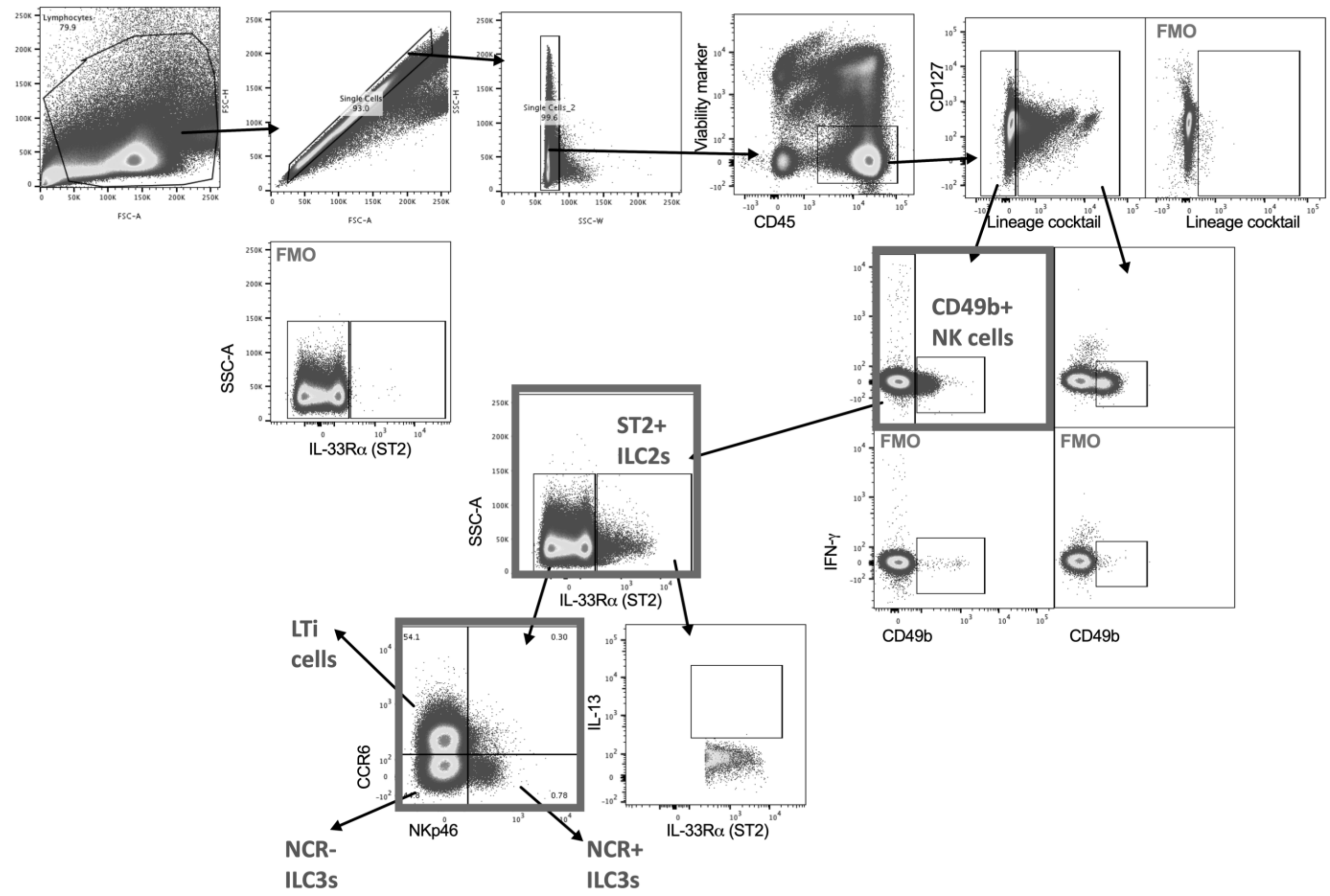

Figure S1. Gating strategy for analysis of innate lymphoid cells. Analysis of spleen and lung innate lymphoid cells (ILC) in BALB/c mice using Fluorescence Minus One (FMOs) [22]. Lymphocytes were gated using the forward scatter/side scatter (FSC/SSC) method, followed by gating on single cells using FSC-A/FSC-H. Gating was then applied on live CD45+ leukocytes for further identification. To exclude mature hematopoietic lineages, a commercially available lineage antibody cocktail was used to gate out CD3e+ T cells, CD45R/B220+ B cells, CD11b+ myeloid cells, Ly-6G/C+ granulocytes, and Ter-119+ erythroid cells. Lineage-negative CD45+ cells were then analyzed to identify ILC subsets. CD49b and IFNγ markers were used to define NK cells expressing IFNγ. IL-33Ra and IL-13 markers were used on CD49b- cells to identify IL-13-expressing ST2+ILC2. NKp46 and CCR6 markers were applied to CD49b- IL-33Ra- mononuclear cells to identify LTi cells, NCR- ILC3 and NCR+ ILC3.
