# Supplementary material for: BCG Vaccine-Induced Innate and Adaptive Pulmonary Immunity Correlating with Protective Efficacy Against Mycobacterium tuberculosis in the Lungs
Source: Vaccines (Basel). 2025 Aug 19;13(8):876. doi: 10.3390/vaccines13080876 (PMC12389847; doi:10.3390/vaccines13080876)
Supplement: Supplementary file 1 [file vaccines-13-00876-s001.zip › Figure S2.pdf]

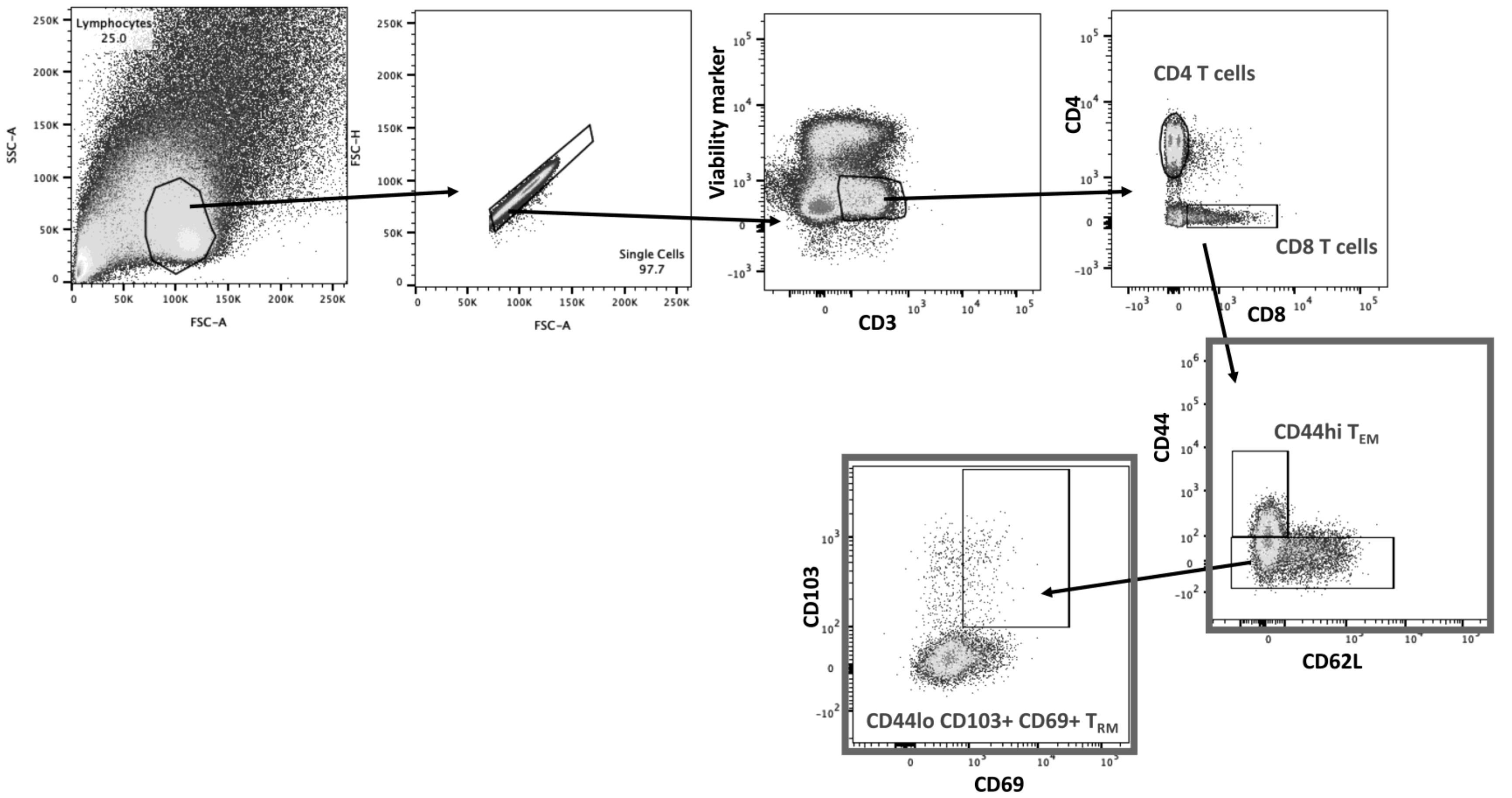

Figure S2. Gating strategy for analysis of memory T cells. Analysis of spleen and lung memory T cells in BALB/c mice. Lymphocytes were gated using the forward scatter/side scatter (FSC/SSC) method, followed by gating on single cells using FSC-A/FSC-H. Gating was then applied on live CD3+ cells for further identification. CD4 and CD8 markers were gated on CD3+ cells to identify helper and cytotoxic T cells respectively. For both CD4 and CD8 T cells, CD44 and CD62L markers were used to identify CD44hi effector memory T cells. Cells negative for the CD44 marker were then gated using CD103 and CD69 to identify CD44lo CD103+ CD69+ resident memory T cells.
