# Supplementary material for: BCG Vaccine-Induced Innate and Adaptive Pulmonary Immunity Correlating with Protective Efficacy Against Mycobacterium tuberculosis in the Lungs
Source: Vaccines (Basel). 2025 Aug 19;13(8):876. doi: 10.3390/vaccines13080876 (PMC12389847; doi:10.3390/vaccines13080876)
Supplement: Supplementary file 1 [file vaccines-13-00876-s001.zip › Figure S3.pdf]

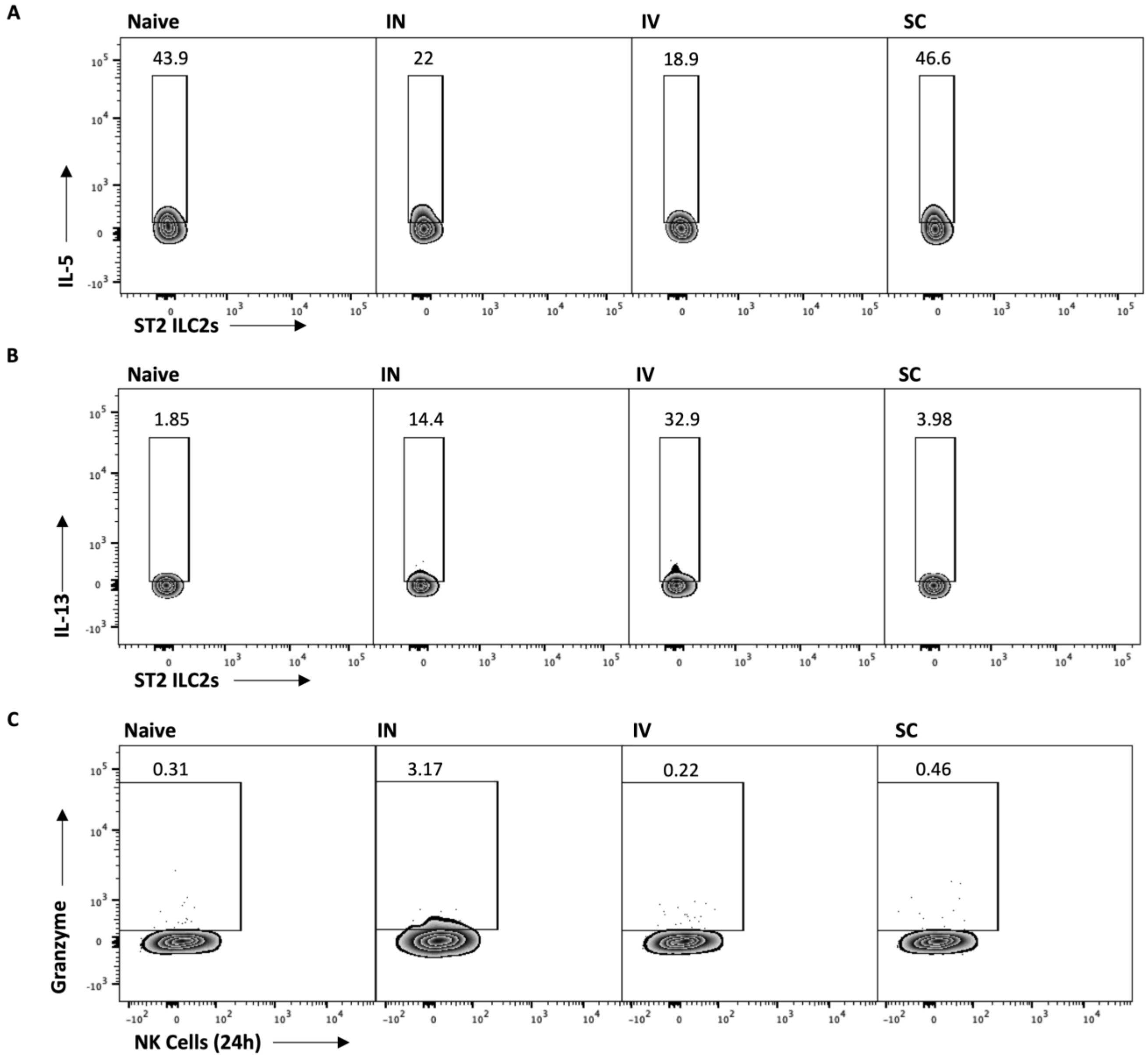

Figure S3. Flow cytometry data plots illustrating the expression of (A) IL-5, (B) IL-13, and (C) granzyme by distinct ILC subpopulations that are representative of ILC characterized in this study.
